# Lupus hands mimicking psoriatic arthritis

**DOI:** 10.2478/rir-2023-0031

**Published:** 2023-12-19

**Authors:** Lidan Zhao, Fengchun Zhang

**Affiliations:** Department of Rheumatology and Clinical Immunology, Peking Union Medical College Hospital, Chinese Academy of Medical Sciences & Peking Union Medical College, Beijing, 100730, China; National Clinical Research Center for Dermatologic and Immunologic Diseases (NCRC-DID), Beijing, 100730, China

**Keywords:** erosive arthritis, • systemic lupus erythematosus

## Abstract

We report a case of a 68-year-old woman with chronic and severely destructive arthritis for 8 years with imaging features mimicking psoriatic arthritis (PsA) but serological evidence of systemic lupus erythematosus. Both the lupus panniculitis–like rash and the presence of interstitial lung disease were considered manifestations of systemic involvement of SLE.

A 68-year-old woman visited the outpatient clinic of Peking Union Medical College Hospital with the chief complaint of pain and deformity in digits of both hands. For more than 8 years, she had suffered from chronic non-severe arthralgia and small hand joints swelling, which mainly involved the distal interphalangeal (DIP) joints with gradually developing deformity of “swan neck” in several fingers of both hands (Figure 1A). She had not searched for medical assistance but self-treated with chinese traditional medicine. For the past 3 months, her arthralgia aggravated and spread to shoulders, knees, and temporomandibular joints with newly occurred erythema nodosa–like skin lesions on distal cruris (Figure 1C). Neither psoriasis-like rash nor nail lesions were found, and the consultation from a dermatologist also excluded psoriasis and stated the possibility of stasis dermatitis or erythema nodosa. The past history of the patient is non-remarkable. Laboratory examinations showed an elevated erythrocyte sedimentation rate (ESR) (40 mm/h, reference: 0–20 mm/h) and C-reactive protein (CRP) level (8.64 mg/L, reference: <3 mg/L). Positive antinuclear antibody (ANA) (titer: 1:80, homogeneous and cytoplasmic pattern), anti–double-stranded DNA (chemiluminescence immunoassay: 69.1 IU/mL, reference: <24 IU/mL), anti-Ro52, and low-titer anti–Jo-1 were identified with hypocomplement-emia (complement 3 0.64 g/L, reference: 0.73–1.46 g/L) and slightly elevated IgG (17.31 g/L, 7–17 g/L), IgA (5.97 g/L, reference: 0.7–4 g/L) and IgM (6.16g/L, reference: 0.4–2.3 g/L). But rheumatoid arthritis (RA)–related antibodies including rheumatoid factor (RF), anti–cyclic citrullinated peptide (a-CCP), antikeratin antibody, anti–perinuclear factor, and anti–mutated citrullinated vimentin were all negative. Anti–phospholipid antibody, anti–neutrophil cytoplasmic autoantibody, creatine kinase, and thyroid function were all normal. The hand X-ray showed multiple bone erosions, subluxation, joint space narrowing, and multiple distal phalanx absorption (Figure 1B). A chest computer tomography (CT) showed the enlargement of mediastinal lymph nodes, pulmonary bulla, and interstitial fibrosis in the base of bilateral lungs (Figure 2). According to 2019 ACR/EULAR SLE classification criteria, the patient was diagnosed with SLE and prescribed prednisone 40 mg/day, hydroxychloroquine 0.2 twice a day, and *Tripterygium wilfordii* 20 mg three times daily with calcium tablets and vitamin D.

**Figure 1:**
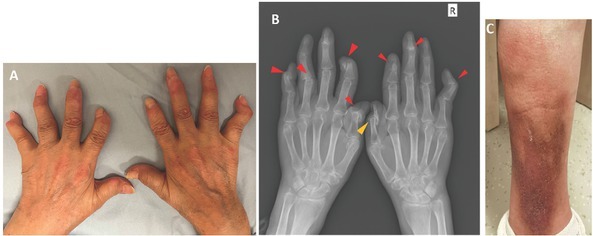
Picture (A) and X-ray image (B) of the patient’s hands showing deformities, destructive arthritis, multiple subluxation, and distal phalanx absorption (red arrows) with “pencil-in-cup” signs observed in the DIP joints of right thumb (yellow arrow). (C) Picture of the erythema nodosa–like rash in the patient’s lower limbs. Abbreviation: DIP, distal interphalangeal.

**Figure 2:**
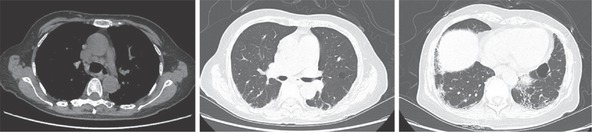
CT showing the enlargement of mediastinal lymph nodes, pulmonary bulla, and interstitial fibrosis in the base of bilateral lungs. Abbreviation: CT, computer tomography.

## Discussion

Arthralgia and arthritis are quite common in SLE but generally considered non-erosive with less need for aggressive treatment. The well-known Jaccoud arthropathy manifests as joint deformity like those in RA, but usually shows no erosions on imaging examination, implicating the existence of tendinitis other than synovitis. RA-like erosive arthritis (EA) with imaging evidence in lupus patients substantially potentiates the diagnosis of “rhupus,” which can be ascertained if both RA and SLE classification criteria are satisfied concurrently. Therefore, rhupus can be considered an overlapping phenotype of SLE and RA. The reported prevalence of rhupus among SLE patients varies from 1.4% to 9.7%, according to recent studies,^[1,2]^ whereas the occurrence of EA in SLE might still be underestimated. With the wide application of sensitive imaging tools (e.g. ultrasound, CT, and magnetic resonance), EA is identified in more SLE patients than previously considered. It is reported that among those SLE patients with joint involvement, EA may exceed 30%.^[3,4]^ Researchers attempted to discover biomarkers associated with EA in SLE, and the suggested predictors or indicators include anti–citrullinated protein antibodies (ACPA),^[5,6]^ anti– carbamylated proteins^[7]^ in serum, elevated IL-17 in synovial fluid,^[8]^ and amount of *Porphyromonas gingivalis* in the tongue biofilm.^[9]^

As for this patient, EA started from DIP and predominantly involved DIP but neither met the classification criteria of RA (2010 ACR/EULAR criteria) nor showed any RA-related autoantibodies in serum. The features of joint erosion and bone destruction on the X-ray mimic those of psoriatic arthritis (PsA) with phalangeal acro-osteolysis and the “pencil-in-cup” sign.^[10]^ Coexistence of osteolysis and osteohyperplasia (the base of DIP joints of the right thumb) in hand joints of the patient provoked the suspicion of PsA, whereas careful examinations found neither psoriatic rash nor nail disease, and serologic examinations indicated the probability of SLE. She met the 2019 ACR/EULAR SLE classification criteria,^[11]^ with a total score of 15 (6 for joint involvement, 6 for positive anti-dsDNA, and 3 for low C3); thus, the diagnosis of SLE was made. In addition, the rash in her lower limbs was erythema nodosa–like, and we were inclined to attribute it to lupus panniculitis (Figure 1C). Unfortunately, a skin biopsy has not been performed to verify the conjecture due to the patient’s unwillingness. The chest CT scan of the patient presented mild subpleural interstitial lung disease in the bases of both lungs, which might be better explained by lupus, instead of psoriasis. As we know, elderly-onset lupus is more insidious and is different from younger patients with arthritis, sicca syndrome, and lung disease being more common, whereas lupus nephritis being less common, with which our patients was consistent.

Finally, although low titer of anti–Jo-1 was detected in the serum, the patient showed neither symptoms or signs of muscle involvement nor the characteristic rash of dermatomyositis, and her creatine kinase level was normal. Actually, low titer anti-Jo-1 can be occasionally found in lupus patients with the application of digital liquid chip method (DLCM) either due to the inherent complex autoantibody spectrum in SLE or due to this sensitive detection method. Based on monolithic analysis, the patient was diagnosed with SLE with EA and interstitial lung disease, and steroid and *Tripterygium wilfordii* were prescribed to control pulmonary fibrosis and arthritis with a long-term follow-up programmed for tracing changes of her symptoms.
